# Cancer-derived exosomal lncRNA SNHG3 promotes the metastasis of colorectal cancer through hnRNPC-mediating RNA stability of β-catenin

**DOI:** 10.7150/ijbs.88313

**Published:** 2024-04-08

**Authors:** Ling Huang, Guang Yang, Yanfei Shao, Jing Sun, Xiao Yang, Hiju Hong, Batuer Aikemu, Galiya Yesseyeva, Shuchun Li, Chengsheng Ding, Xiaodong Fan, Sen Zhang, Junjun Ma, Minhua Zheng

**Affiliations:** 1 Department of General Surgery, Ruijin Hospital, Shanghai Jiao Tong University School of Medicine, Shanghai, China.; 2 Shanghai Minimally Invasive Surgery Center, Ruijin Hospital, Shanghai Jiao Tong University School of Medicine, Shanghai, China.; 3 Shanghai Institute of Digestive Surgery, Ruijin Hospital, Shanghai Jiao Tong University School of Medicine, Shanghai, China.

**Keywords:** colorectal cancer, metastasis, exosome, lncRNA

## Abstract

Metastasis is the leading cause of death in colorectal cancer (CRC) patients. By mediating intercellular communication, exosomes exhibit considerable value in regulating tumor metastasis. Long non-coding RNAs (lncRNAs) are abundant in exosomes and participate in regulating tumor progression. However, it is poorly understood how the cancer-secreted exosomal lncRNAs affect CRC proliferation and metastasis. Here, by analyzing the public databases we identified a lncRNA SNHG3 and demonstrated that SNHG3 was delivered through CRC cells-derived exosomes to promote metastasis in CRC. Mechanistically, exosomal SNHG3 was internalized by CRC cells and afterward upregulated the expression of β-catenin by facilitating the intranuclear transport of hnRNPC. Consequently, the RNA stability of β-catenin was enhanced which led to the activation of EMT and metastasis of CRC cells. Our findings expand the oncogenic mechanisms of exosomal SNHG3 and identify it as a diagnostic marker for CRC.

## Introduction

Colorectal cancer (CRC) is one of the most malignant diseases, which ranked the fourth leading cause of death around the world 1, 2. Despite the improvement of therapeutic effects in the past few decades, the survival rate of CRC patients, especially those with advanced stages is far from satisfactory. The poor prognosis of CRC is largely owing to its high tendency to relapse and metastasize 3, 4. Thus, an exploration into the mechanism of the progression and metastasis of CRC is required to refine early screening approaches and therapeutic strategies, which may promise a better prognosis for CRC patients.

Exosomes are a subpopulation of extracellular vesicles with a diameter of 30-150nm that can be released by a variety of cell types. In addition, they stably exist in several kinds of body fluids, such as serum, milk, bile, urine, and cerebrospinal fluid 5. Carrying multifarious cargos from parental cells, exosomes were reported to play a vital role in mediating intercellular communications. Increasing evidence has revealed the direct involvement of exosomes in promoting the progression of tumors 6-9. By transferring functional molecules, cancer cells-derived exosomes help to shape the biological characteristics of recipient cells and create a suitable microenvironment for the proliferation and metastasis of tumors, and even assist them to escape from the toxicity of anticarcinogens. The characteristics of wide distribution and stable existence in body fluids of exosomes make them one of the optimal candidates in fluid biopsy, which has attracted increasing interest as a method of tumor early detection 10, 11.

Long non-coding RNAs (lncRNAs), which consist of more than 200 nucleotides are the major class of noncoding transcripts. The complicated mechanisms of lncRNAs in regulating the initiation and progression of tumors have not been completely unveiled as lncRNAs are much more diverse than other noncoding RNAs. Two of the most commonly seen functions of lncRNAs are epigenetic regulation and nuclear structure construction, like m6A-modification can be regulated by a newly described lncRNA Lung Cancer Associated Transcript 3 (LCAT3) 12. Exosomal lncRNAs have been indicated to play a part in educating the recipient cells and promoting tumor progression 13, 14. However, their regulation patterns in CRC remain poorly clarified and there is still a lack of efficient exosomal-biomarkers for the early detection of CRC. Here, by screening public databases we speculated that exosomal lncRNA SNHG3 may be important to the progression of CRC. Thus, we investigated the oncogenic role of SNHG3 in CRC by conducting *in vitro* and *in vivo* experiments and revealed that exosomal SNHG3 can promote the migration of CRC through upregulating β-catenin and activating the EMT pathway. Our study confirmed the significance of exosomal SNHG3 in the development and diagnosis of CRC.

## Material and methods

### Antibodies and reagents

Primary antibodies including: hnRNPC (Mouse, catalog number ab10294; Rabbit, catalog number ab133607), TSG101 (Mouse, catalog number ab83) and CD63 (Mouse, catalog number ab59479) were obtained from Abcam (Cambridge, England); β-catenin (Rabbit, catalog number 8480), Grp94 (Rabbit, catalog number 20292), Snail (Rabbit, catalog number 3879) and Slug (Rabbit, catalog number 9585) were purchased from Cell Signaling Technology (Massachusetts, USA); E-cadherin (Mouse, catalog number 60335-1-Ig) and GAPDH (Mouse, catalog number 60004-1-Ig) were from Proteintech (Wuhan, China); Vimentin (Rabbit, catalog number A11952) and N-cadherin (Rabbit, catalog number A3045) were from ABclonal (Wuhan, China). Mg132 was purchased from Meilunbio (Dalian, China), XAV-939 was purchased from Target Molecule Corp. (Massachusetts, USA).

### Cell culture and transfection

The human CRC cell lines RKO and HCT 116 were acquired from the American Type Culture Collection (ATCC, Manassas, VA). The lentiviruses were constructed by Genechem Inc. (Shanghai, China), to establish cell lines stably overexpressing and silencing SNHG3, respectively. All the cells were incubated with RPMI-1640 medium (Meilunbio) supplemented with 10% fetal bovine serum (FBS, Gibco, Grand Island, NY, USA) and 1% penicillin-streptomycin (New Cell & Molecular Biotech, Suzhou, China) and placed in 37 °C incubator containing 5% CO_2_. Sequences of sh-SNHG3 lentivirus were as follows: sense-1 5′-GGGCACTTCGTAAGGTTTAAA-3′, antisense-1 5'-TTTAAACCTTACGAAGTGCCC-3'; sense-2 5′-GGTTGAGTGCAAGATGAGTTA-3′, antisense-2 5'-TAACTCATCTTGCACTCAACC-3'.

### Bio information analysis

TCGA-COAD and TCGA-READ datasets with the corresponding clinical data (UCSC) were downloaded to explore the differentially expressed (DE) lncRNAs in CRC. CRC_lncRNAs dataset was obtained from exoRBase 1.0 to detect the differential expression of lncRNAs in serum-derived exosomes. The R package “limma” was used to analyze the DE lncRNAs between normal and tumor samples, under the setting of false discovery rate (FDR) <0.01 and |log fold change (logFC)| >1. A web tool named “Venny” was applied to create the Venn diagram visualizing the DE lncRNAs.

### Exosome isolation and identification

The exosomes were isolated according to the ultracentrifugation steps 15. Briefly, the cell supernatant was collected and passed through a 0.22 μm filter. After which the supernatant was centrifugated at 10 000g for 30 min and then subjected to ultracentrifugation at 100 000g for 70 min. To remove the contaminating proteins, the pellet was resuspended with PBS and centrifugated once more at 100 000g for 70 min. The exosomes were examined by Transmission Electron Microscopy and western blotting to detect the exosomal marker.

Serum exosomes were isolated using the Total Exosome Isolation kit (#4484450, Thermo Scientific) as described in the manufacturer's instructions.

DiI dye (MB4240, Meilunbio) was used to label and track exosomes. The isolated exosomes (20 μg/ml) were incubated with DiI for 1 h under the condition of cell culture. Before being added to the culture medium and incubated with CRC cells, the labeled exosomes were washed twice with PBS and then isolated.

### Cell viability, cell migration, and wound healing assays

Cell viability assays, including colony formation and CCK-8 assay, were conducted as previously described 16. To perform cell migration assay, 6 × 10^4^ HCT 116 or 1.5 × 10^5^ RKO cells suspended with 300 μl serum-free culture medium were seeded in the upper chambers of Transwell (8 μm for 24-well plate; Corning Costar, NY, USA). 700 μl culture medium with 15% FBS was added to the lower chambers. After 24h incubation for HCT 116 and 96h for RKO, the lower side of the upper chambers were washed using PBS, fixed with 4% formaldehyde, stained with 0.25% crystal violet, and analyzed with an optical microscope.

For wound healing assay, adequate CRC cells were placed into 12-well dishes and made sure the confluence could reach 80-90%. Wounds were scratched with sterilized one-milliliter pipette tips, after which the cell debris was washed with PBS. The observation was conducted at 0h, 24h, and 48h. and each time five randomly-selected areas of the wounds were photographed under an optical microscope.

### RNA extraction and quantitative real-time PCR (qPCR)

Total RNA extraction and qPCR were carried out as previously mentioned 17. Sequences of SNHG3 primer: sense 5'-TGGGACTGAAGGGGGATCAT-3', antisense 5'-CTCACCCACCTCCCTCAAAC-3'; sequences of β-catenin primer: sense 5'-AAAGCGGCTGTTAGTCACTGG-3', antisense 5'-CGAGTCATTGCATACTGTCCAT-3'.

### Western blotting

Western blotting was performed as previously described 16. To estimate the distribution and cellular localization of specific proteins, the subcellular protein fractionation kit (#78833; Thermo Scientific, Waltham, MA, USA) was used to extract proteins from nuclear and cytoplasmic fractions, and the assay was conducted according to the manufacturer's instruction.

### RNA pulldown and RNA immunoprecipitation assay

RNA pulldown assay was carried out under the guidance of the instruction provided by the Pierce Magnetic RNA-Protein Pull-Down Kit (#20164, Thermo Scientific). Briefly, Biotin-16-UTP (#11388908910, Sigma-Aldrich, USA) and HiScribe T7 High Yield RNA Synthesis Kit (#E2040S, NEB, USA) were adopted to transcribe specific plasmids into biotin-labeled RNAs, including sense and antisense sequences. Which were then bound to the streptavidin magnetic beads, and incubated overnight with the nuclear fraction obtained by the subcellular protein fractionation kit. The RNA-binding proteins were eluted from the beads and analyzed by western blotting or silver staining.

RNA immunoprecipitation assay was performed following the instruction of the EZ-Magna RIP kit (#17-700, Sigma-Aldrich). Cell lysates were obtained using the RIP lysis buffer, and incubated with the RIP buffer containing antibody-conjugated magnetic beads overnight. The immunoprecipitated RNAs were extracted with TRIzol reagent and analyzed by qPCR.

### Immunofluorescence

Cell suspensions were added to a 24-well plate covered with sterile coverslips. After adherence, the culture plate was collected and every well with a slide was washed with PBS and then fixed with icy 4% formaldehyde at 4 °C for 20 min. Then, we used 0.2 % Triton-X 100 to penetrate the cell membrane at 4 °C for 10 min. After being blocked with 0.2 % BSA, cells were incubated with primary antibody dissolved in 0.2 % BSA at 4 °C overnight. After which, the slides were washed with PBS and incubated with diluted fluorescent secondary antibody for 1.5 h at room temperature. During the final step, the slides were mounted with an antifade mounting solution containing 4,6-diamidino- 2-phenylindole (DAPI; P36935, Invitrogen). Cells were observed and photographed using fluorescence microscopy (TS2R-FL, Nikon, Japan).

### Xenograft model

Subcutaneous xenograft model was constructed to analyze the proliferative capacity of CRC cells in vivo. A total of 5 × 10^6^ HCT 116 cells in 100 μl PBS were injected subcutaneously into the armpits of nude mice. Tumor volume (V) was determined by measuring the length and width of the tumor and using the formula V = (width*width*length) / 2. Four weeks after the injection, all the nude mice were sacrificed and the tumor grafts were resected completely which were then pretreated for further qPCR or western blotting to detect the expression level of specific molecules.

The peritoneal metastatic xenograft model was constructed as previously described 18, to estimate the metastatic capacity of CRC cells. Before injection, stable cell lines were infected with luciferase-expressing lentivirus. A total of 5 × 10^6^ HCT 116 cells in 200 μl PBS were injected intraperitoneally into the nude mice. Before being sacrificed, tumor distribution was assessed by bioluminescence imaging (Caliper Life Sciences, USA) in the fourth week.

### Statistical analysis

All the data were calculated with SPSS statistics 23 and GraphPad Prism 6.02, and are presented as the mean ± standard deviations. Two-tailed Student's *t*-test and one-way ANOVA were adopted for parametric variables and the chi-square test was applied for categorical variables. Differences with a *P* value < 0.05 were considered statistical significance.

## Results

### SNHG3 was upregulated in serum-derived exosomes and tumor tissues of CRC patients

**Figure 1:**
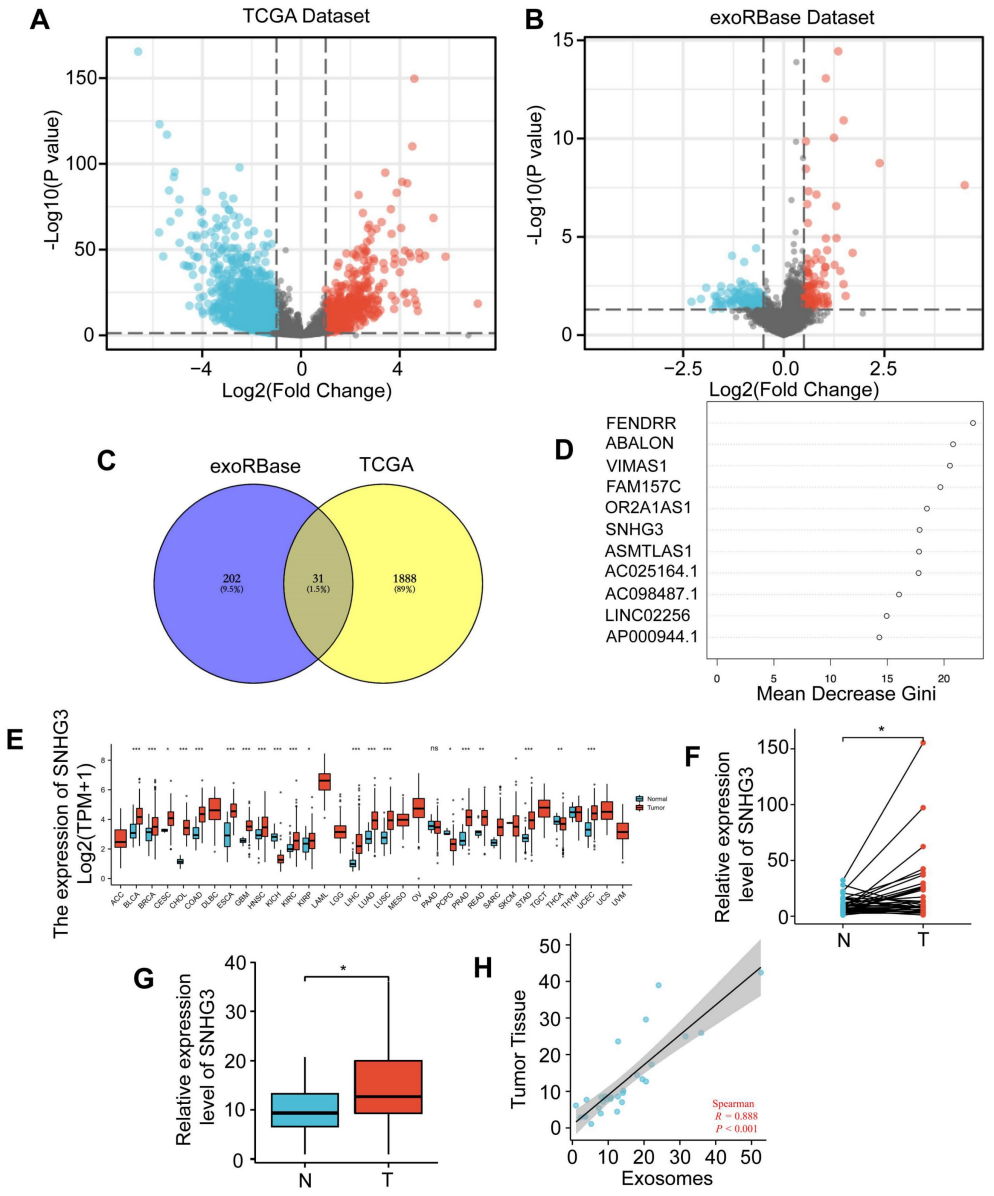
**SNHG3 was upregulated in serum-derived exosomes and tumor tissues of CRC patients. A** Differentially expressed (DE) lncRNAs were explored using the TCGA dataset. **B** Exosomal DE lncRNAs were explored using exoRBase dataset 1.0. **C** Venn plot showing the intersection of these two groups of DE lncRNAs. **D** Random Forest was applied to rank the importance of the 31 DE lncRNAs. **E** GEPIA indicated the upregulation of SNHG3 in multiple cancers. **F** and **G** The level of SNHG3 has confirmed an increase in CRC tumor tissues and serum-derived exosomes of CRC patients. H The expression level of SNHG3 was positively correlated in serum-derived exosomes and the corresponding tumor tissues was of CRC patients. The error bar represents the mean ± SD of three independent experiments. *p < 0.05, **p < 0.01, ***p < 0.001.

The CRC datasets in TCGA were downloaded to explore the differentially expressed lncRNAs between tumor and normal tissues. Totally we found 1241 upregulated and 677 downregulated lncRNAs in CRC which were visualized by a volcano plot and shown in Fig. 1A. To narrow down the candidates and screen out the ones that can be secreted and work on distant organs through exosomes, we also analyzed the CRC lncRNA datasets in exoRBase 1.0 by comparing the serum-derived exosomes from CRC patients and normal participants (Fig. 1B). Then an intersection was taken for these two groups of DE lncRNAs and visualized by a Venn diagram (Fig. 1C). According to the result, 31 lncRNAs were selected based on their expression variance both in tumor tissues and serum-derived exosomes. For further analysis, the random forest method was adopted to assess the importance of these lncRNAs by calculating and ranking the mean decrease in the Gini coefficient (Fig. 1D). Based on the result, the most important lncRNAs were FENDRR, ABALON, VIMAS1, FAM157C, OR2A1AS1, SNHG3, ASMTLAS1, AC025164.1, AC098487.1, and LINC02256. To further narrow down the list, we analyzed the differential expressions of these ten lncRNAs in the pan-cancer database of TCGA. As shown, SNHG3 was most significantly upregulated in multiple cancers compared to other 9 lncRNAs (Fig. 1E and S1). SNHG3 belongs to a lncRNA family known as the small nucleolar RNA host genes (SNHGs) family, of which most members have been reported to participate in the process of tumor progression 19-21. Thus, we selected SNHG3 as the target of our following research. To confirm the differential expression of SNHG3 in CRC, a cohort of 36 pairs of CRC tumor tissues and paired normal tissues (at a distance of at least 5 cm from the tumor edge) from our center were collected and the levels of SNHG3 were analyzed. It could be seen from Fig. 1F that SNHG3 was overexpressed in CRC tumor tissues. Consistent with that, the SNHG3 expression levels in 23 cases of serum-derived exosomes from CRC patients were significantly higher than those in 19 cases of normal participants (Fig. 1G). Moreover, it was observed that the expression levels of SNHG3 in serum-derived exosomes were also positively correlated with those in tumor tissues (Fig. 1H). Thus, we speculated that SNHG3 may play a critical role during the progression of CRC.

### SNHG3 was secreted through exosomes and activated the EMT pathway in recipient CRC cells

**Figure 2:**
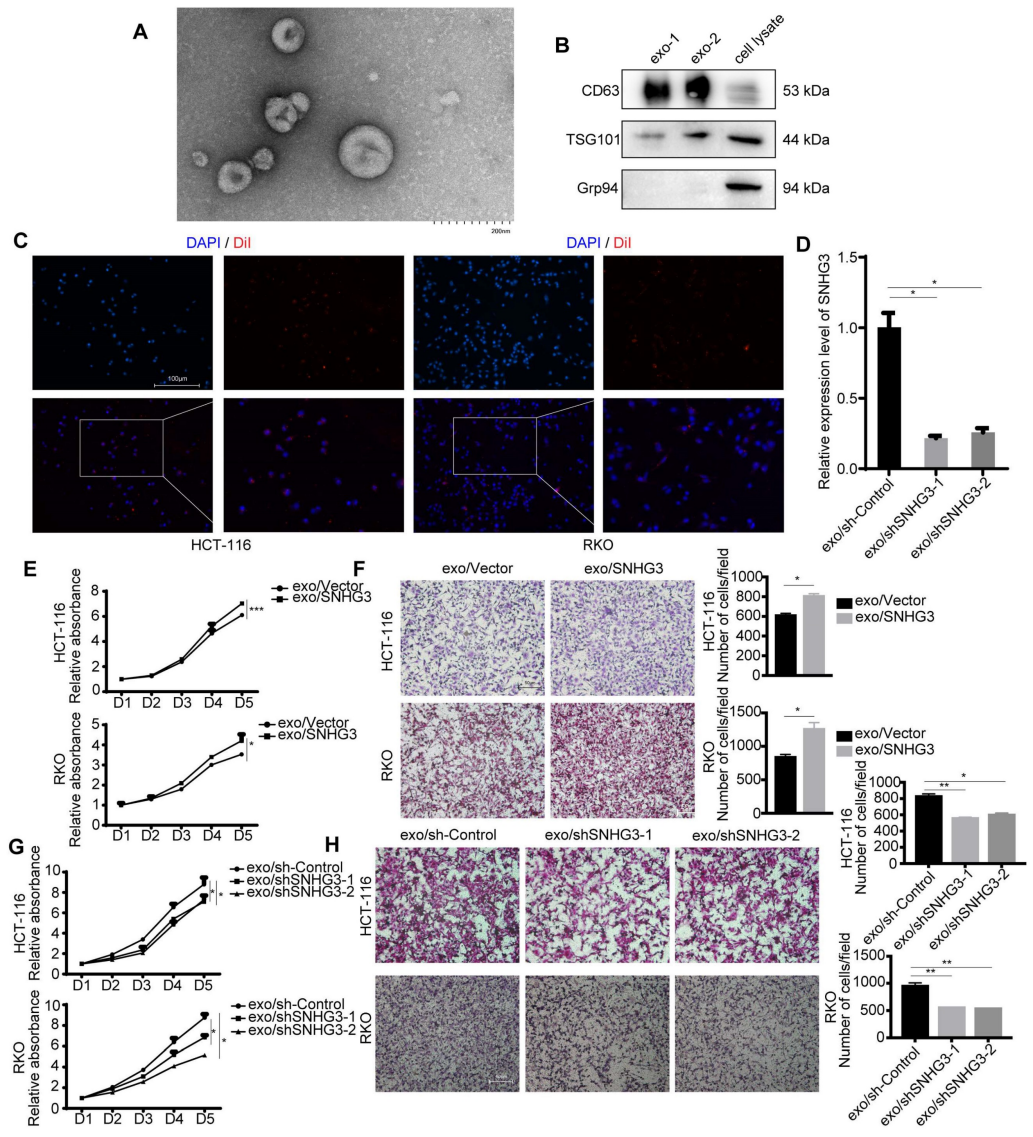
**Exosomal SNHG3 promotes the proliferation and migration of CRC cells. A** TEM image of purified exosomes. Scale bar=200 nm. **B** The exosome biomarkers (CD63 and TSG101) were assessed by Western blot, and Grp94 served as the internal control of whole-cell lysates. **C** The internalization of DiI-labeled exosomes secreting by HCT 116 cell lines was observed using an immunofluorescent microscope. Scale bar=200 μm. **D** Real-time PCR was applied to analyze the expression levels of exosomal SNHG3 in different groups of stably transfected RKO cell lines. **E** and **G** CCK-8 assay and **F** and **H** Transwell assay were performed to determine the cell viability and migration of wt HCT 116 and RKO cells which were cocultured with exosomes derived from different groups of RKO cells. The error bar represents the mean ± SD of three independent experiments. *p < 0.05, **p < 0.01, ***p < 0.001.

The existence of exosomes was characterized by TEM and immunoblotting (Fig. 2A, 2B and S2A). Two of the commonly used exosomal surface biomarker, CD63, and TSG101 were detected. Grp94 (glucose-regulated protein 94), which is a member of the HSP90 family and resides in the ER (endoplasmic reticulum) was chosen to be the negative control 22. To visualize the absorbance of exosomes by HCT 116 and RKO cell lines, we applied a membrane dye DiI to label and track exosomes derived from HCT 116 or RKO cell lines which could be observed by a fluorescence microscope (Fig. 2C and S2B). The expression level of SNHG3 in CRC cell-derived exosomes was determined, and the results indicated that SNHG3 could be packed into exosomes and its expression level was altered in accordance with the parental cells (Fig. 2D). Then we cocultured RKO and HCT 116 wild-type cell lines (wt RKO and wt HCT 116) with exosomes collected from the culture medium of HCT 116 cell lines of which SNHG3 was stably upregulated or downregulated, and analyzed the proliferative and metastatic ability of the recipient cells. As shown in Fig. 2E and F the exosomes with high SNHG3 levels could promote the proliferation and metastasis of wt RKO and wt HCT 116 cells, which might be impaired when exosomal SNHG3 was decreased (Fig. 2G and H). We also observed that after being cocultured with high-SNHG3 exosomes, the CRC cells presented a more mesenchymal morphology while the low-SNHG3 exosomes coculture group exhibited a more epithelial morphology compared with the control group (Fig. S2C), which indicated that SNHG3 may activate the epithelial-mesenchymal transition (EMT) to promote the proliferative and metastatic ability of CRC cells. These results suggested that CRC cells could secret SNHG3 through exosomes to promote the proliferation and metastasis of the recipient CRC cells.

### SNHG3 functioned as an oncogene in CRC cells

**Figure 3:**
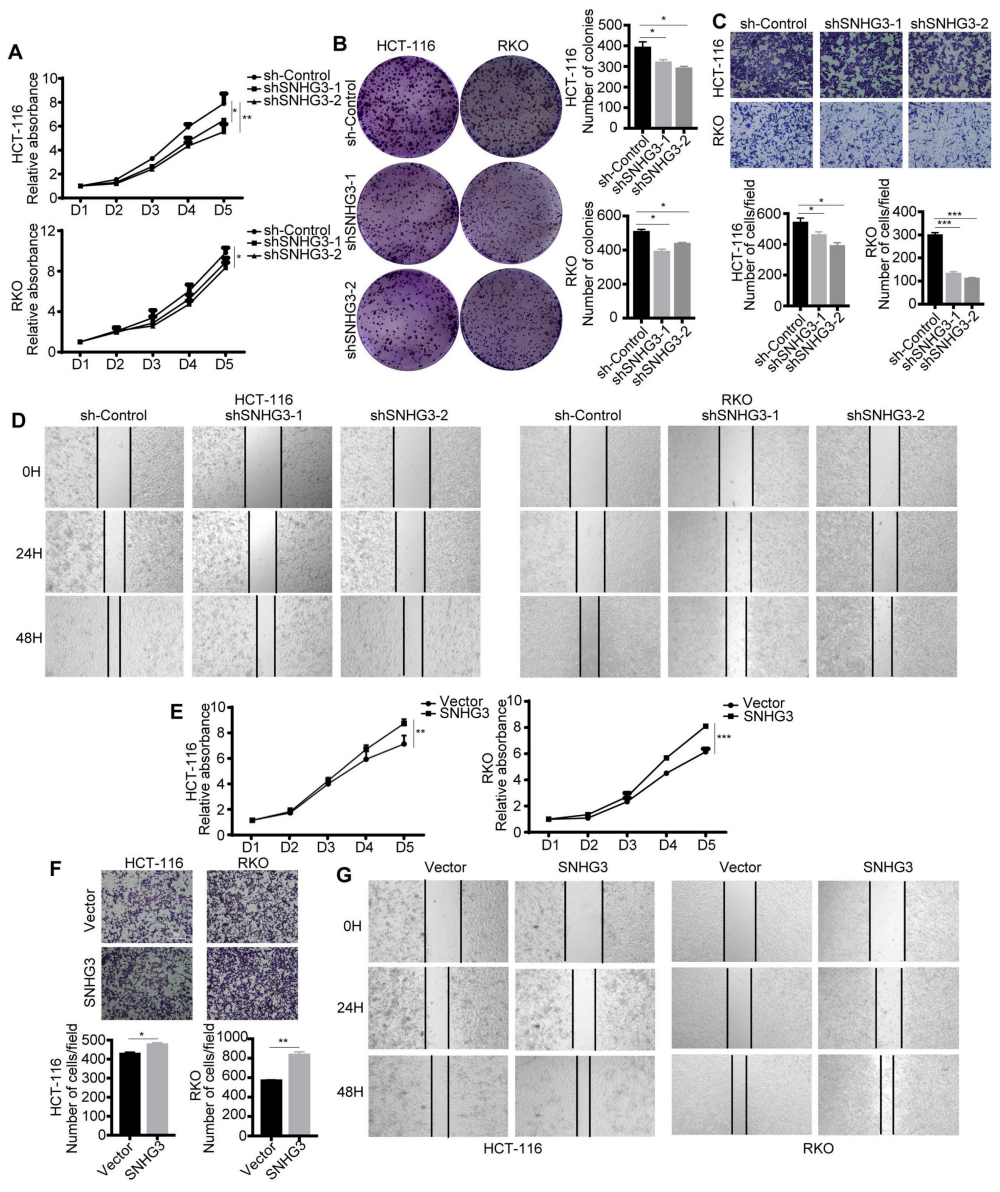
**SNHG3 acts as a promoter during the progression of CRC. A** CCK-8 assay and **B** colony formation assay was performed to assess the proliferation ability of SNHG3-KD HCT 116 and RKO cells. **C** Transwell assay and **D** Wound healing assay were applied to determine the migration ability of SNHG3-KD HCT 116 and RKO cells. **E** CCK-8 assay was performed to assess the proliferative ability of SNHG3-OE HCT 116 and RKO cells. **F** Transwell assay and **G** Wound healing assay was applied to determine cell migration of SNHG3-OE HCT 116 and RKO cells. The error bar represents the mean ± SD of three independent experiments. *p < 0.05, **p < 0.01, ***p < 0.001.

To confirm the biological function of lncRNA SNHG3 and ensure that it was SNHG3 that served as a pivotal factor in CRC cells-derived exosomes modulating the biological characteristics of the recipient CRC cells, we explored the effect of SNHG3 on the proliferation and metastasis of CRC cells. Results of proliferation assays, including CCK-8 and colony formation assay, showed that cell proliferative ability and the number of cell colonies of RKO and HCT 116 cells were decreased when SNHG3 was knocked down (Fig. 3A and B). And the wound healing and cell migration assay illustrated that the decrease of SNHG3 also downregulated the metastatic ability of CRC cells (Fig. 3C and D). On the contrary, overexpressing SNHG3 in RKO and HCT 116 cells facilitated their proliferation and metastasis (Fig. 3E-G).

We further conducted the reverse assay to verify the function of SNHG3, which showed that the effect of overexpressing or decreasing SNHG3 in vitro could be reversed when the expression level of SNHG3 was downregulated or upregulated respectively (Fig. S3A and B). In general, these results suggested that SNHG3 is effective in promoting the proliferation and metastasis of CRC cells.

### SNHG3 activated the EMT pathway to promote the migration of CRC

**Figure 4:**
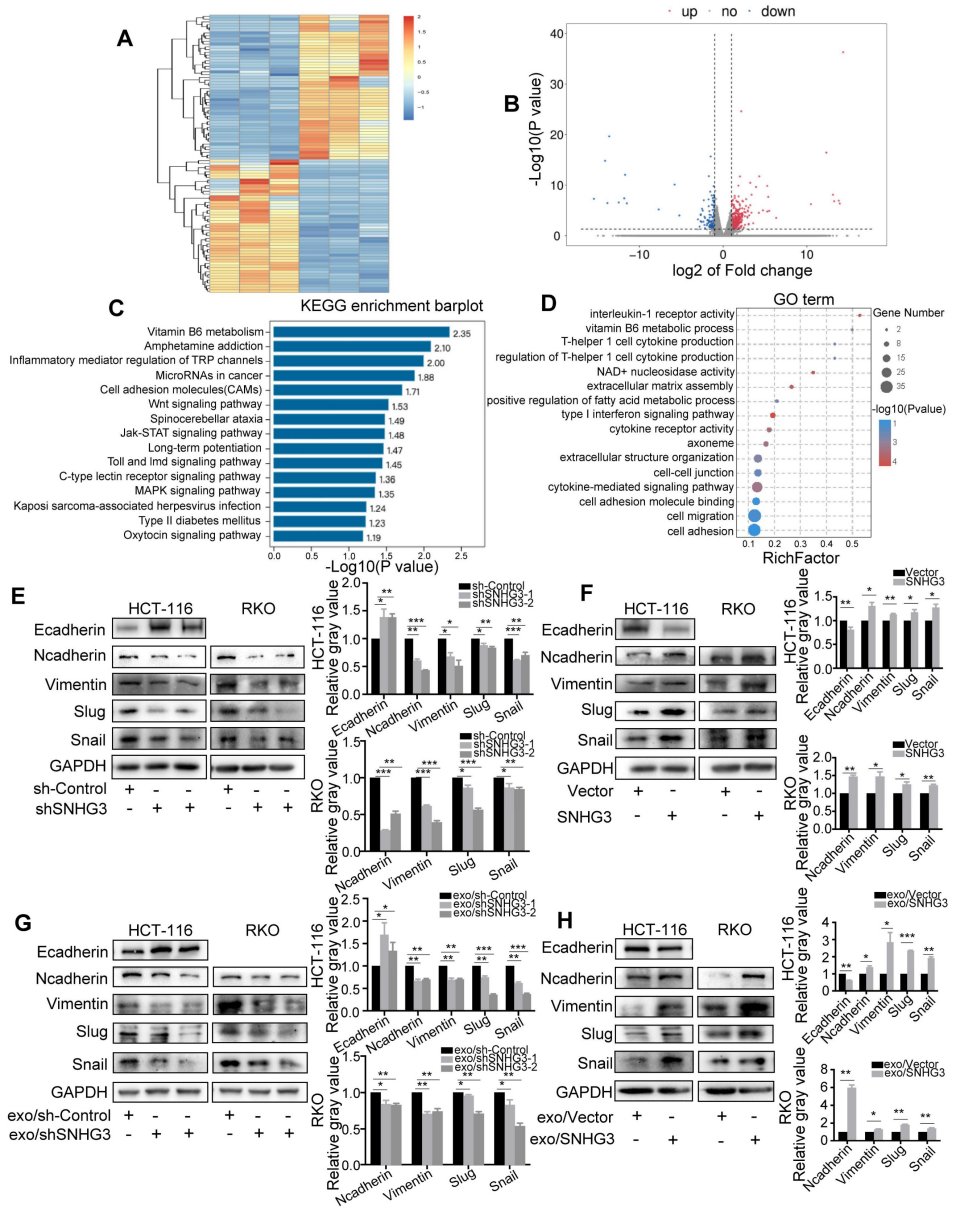
**SNHG3 activated EMT to promote the migration of CRC cells. A** Heatmap diagram and **B** volcano plot of the differentially expressed genes between Control and SNHG3-KD RKO cells (consisting of shSNHG3-1 and shSNHG3-2 groups). **C** KEGG pathway enrichment and **D** GO term analysis of the differential expression genes. **E** and **F** Western blot of EMT markers in Control and SNHG3-KD HCT 116 and RKO cells. **G** and **H** Western blot of EMT markers in wt HCT 116 and RKO cells treated with exosomes derived from stably transfected RKO cells. The error bar represents the mean ± SD of three independent experiments. *p < 0.05, **p < 0.01, ***p < 0.001.

We next performed second-generation sequencing on the total RNA extracted from SNHG3-knockdown (SNHG3-KD) RKO cells to further explored the potential pathways that may be modulated by SNHG3. Differential expression analysis revealed a total of 484 upregulated genes and 114 downregulated genes with statistical significance and was presented by heatmap and a volcano plot (Fig. 4A and B). Following that, we conducted KEGG pathway analysis and GO analysis which was exhibited in Fig. 4C and D.

We found that the depletion of SNHG3 led to the enrichment of 'Cell migration' and 'Cell-cell adhesion' GO terms, and 'Cell adhesion molecules' and 'Wnt signaling pathway', which were consistent with the effect of SNHG3 on the migratory phenotype of CRC cells. We speculated that SNHG3 could activate EMT of CRC cells and therefore performed immunoblotting to evaluate the protein levels of a set of EMT markers to confirm this hypothesis. As shown, we found that the mesenchymal markers including N-cadherin, Vimentin, Snail, and Slug were downregulated and the epithelial marker E-cadherin was upregulated in SNHG3-KD cells (Fig. 4E). Conversely, in SNHG3-overexpressed (SNHG3-OE) groups, normal-SNHG3 exosomes groups and high-SNHG3 exosomes groups, the levels of mesenchymal markers were remarkably increased and the expression of E-cadherin was decreased (Fig. 4F, G and H). Taken together, our observations implied that EMT could be the critical pathway in the process of SNHG3-activated metastasis of CRC cells.

### SNHG3 enhanced the localization of hnRNPC in the nucleus

**Figure 5:**
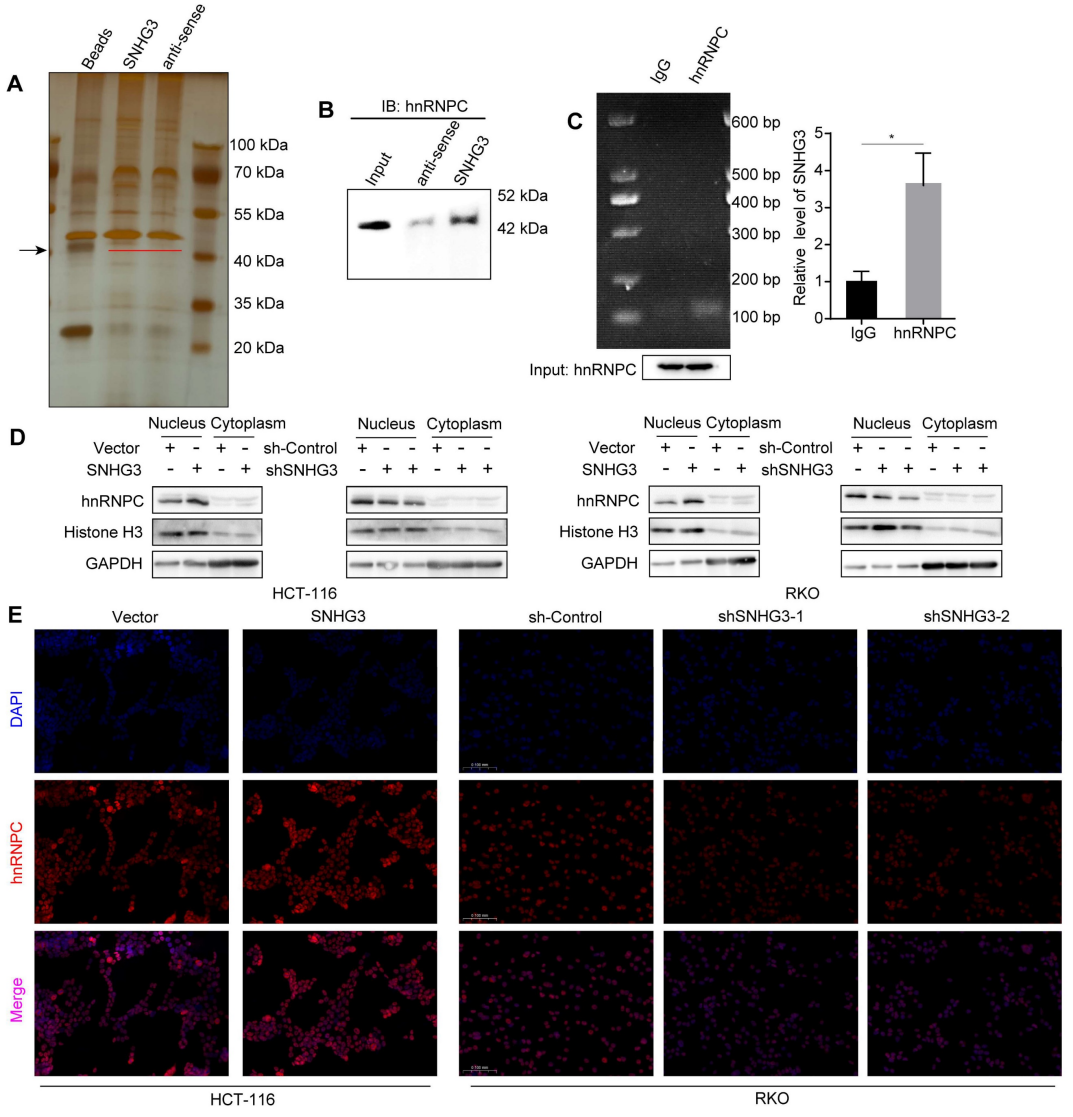
**SNHG3 interacts with hnRNPC and assists it with intranuclear transport. A** Silver-stained SDS-PAGE gel visualized proteins achieved from RNA pulldown by SNHG3 and anti-sense of SNHG3, the lane of beads served as a negative control. The black arrow indicates the band containing differentially expressed proteins. **B** Western blot of the RNA pulldown confirmed that hnRNPC was bound to SNHG3. **C** RNA immunoprecipitation using anti-hnRNPC antibody was carried out upon wt RKO cells. **D** Proteins in the cell nucleus and cytoplasm fractions were extracted and examined by western blot. **E** Immunofluorescence staining exhibited the intracellular localization of hnRNPC in CRC cells. The error bar represents the mean ± SD of three independent experiments. *p < 0.05.

The functions of lncRNA are usually intimately correlated with its subcellular localization. According to the lncATLAS database, SNHG3 exists not only in the cytoplasm but also in the nucleus, and its proportion alters with the change of cell types (Fig. S4A) 23. To verify the distribution of SNHG3 in CRC cell lines, we extracted RNA separately from the cytoplasm and nucleus of RKO and HCT 116 wt cells and quantified the RNA levels using real-time PCR. The results showed a predominant localization of SNHG3 in the nucleus of CRC cells, which suggested that SNHG3 may work with proteins to modulate the expression of downstream molecules (Fig. S4B). Accordingly, we performed RNA pulldown to detect the specific proteins that interacted with SNHG3 and found differentially expressed bands around 40 kDa (Fig. 5A). StarBase and NPInter v4.0 database were applied respectively to predict the proteins that may potentially bind with SNHG3, then the intersection was shown by Venn diagram (Fig. S4C) 24, 25. Among the 17 candidate proteins, hnRNPC is the one with a molecular weight of about 42 kDa and has been reported to interact with lncRNA SNHG1 26 (Table S1). Based on these findings, we hypothesized that hnRNPC was one of the downstream molecules of SNHG3. To confirm the interaction between SNHG3 and hnRNPC, we performed immunoblotting after RNA pulldown, and enrichment of hnRNPC was observed in the sense SNHG3 group (Fig. 5B). In addition, to further verify it we conducted RIP assays and real-time PCR, of which the experimental products were visualized using agarose gel electrophoresis (Fig. 5C). To figure out the effect of SNHG3 on hnRNPC, we conducted immunoblotting and immunofluorescence to assess the distribution of hnRNPC in different cellular fractions. And the results indicated that SNHG3 would facilitate the intranuclear transport of hnRNPC (Fig. 5D, 5E and S4D). Together, these data suggested that SNHG3 may interact with and assist in the intranuclear transport of hnRNPC, which probably boosts the function of hnRNPC to upregulate the migrative ability of CRC cells.

### SNHG3 enhanced the effect of hnRNPC on the RNA stability of β-catenin

**Figure 6:**
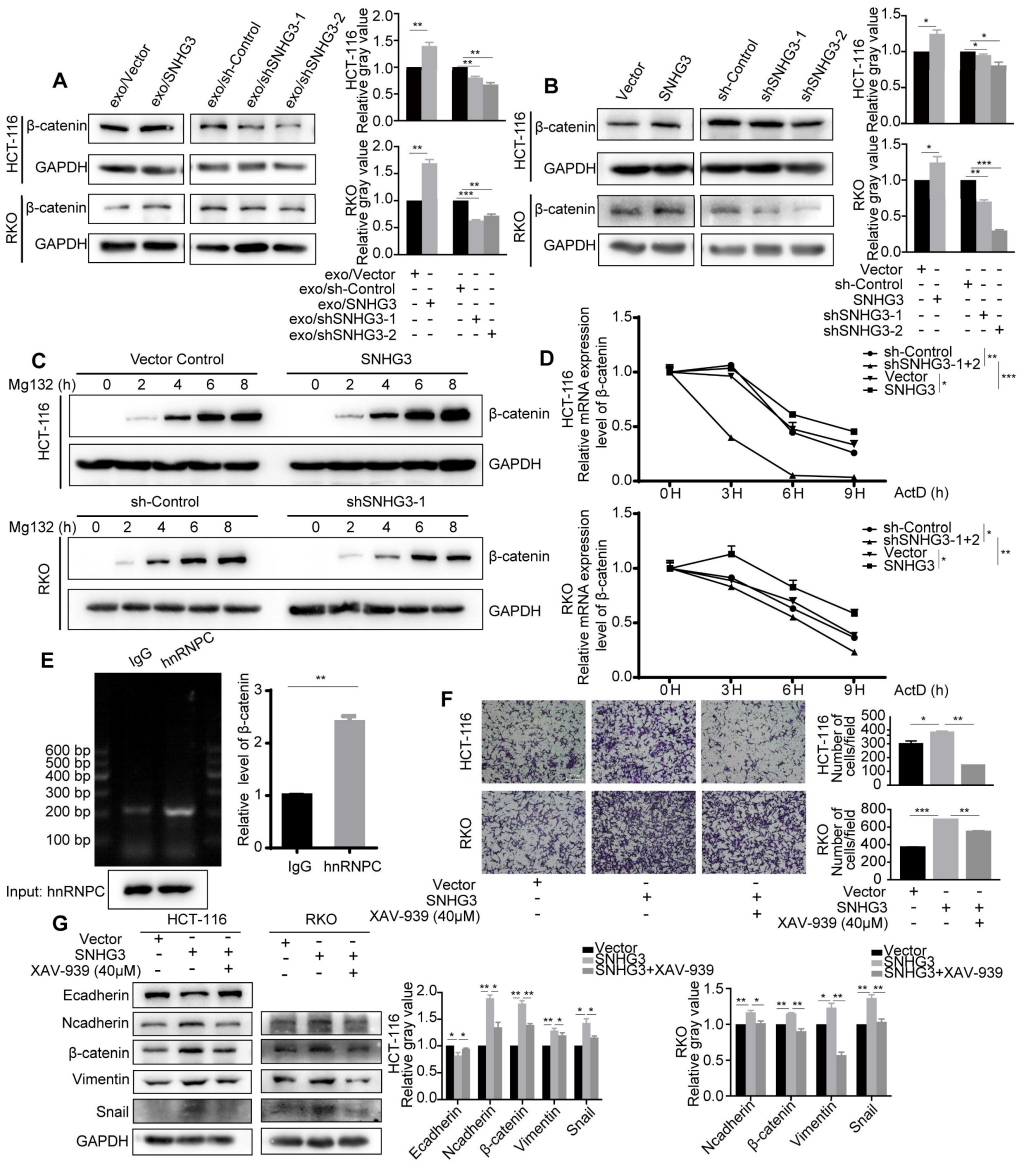
** SNHG3 upregulates β-catenin to activate EMT in CRC cells by enhancing the function of hnRNPC in stabilizing β-catenin transcript. A** and **B** Western blot detected the level of β-catenin in different groups of CRC cells. **C** The expression of β-catenin in cells was determined after being treated with Mg132 at regular intervals. **D** Real-time PCR was applied to detect the level of the β-catenin transcript after the treatment of ActD at regular intervals. **E** RNA immunoprecipitation using anti-hnRNPC antibody was carried out upon wt RKO cells. **F** Transwell assay was used to assess the migration ability of SNHG3-OE HCT 116 and RKO cells after being treated with the β-catenin inhibitor XAV-939. **G** The expression changes of the EMT markers were examined with western blot after the treatment of XAV-939. The error bar represents the mean ± SD of three independent experiments. *p < 0.05, **p < 0.01, ***p < 0.001.

β-catenin is one of the core regulators in the Wnt signaling pathway which has been identified as a crucial pathway during the process of EMT. β-catenin can be tagged by ubiquitin and then sent for degradation. By assessing the protein levels of each group, we found that β-catenin was increased in high-SNHG3 exosomes groups and SNHG3-OE groups, while β-catenin would decrease when SNHG3 was knocked down (Fig. 6A and B). To delve into the detailed mechanism, we firstly applied Mg132, a proteasome inhibitor, to examine the influence of protein degradation on β-catenin. And the result indicated that protein degradation was a less relevant factor during the manipulation of SNHG3 (Fig. 6C). Next, we used Actinomycin D, a transcription inhibitor, to assess the stability of the β-catenin transcript. The RNA levels of β-catenin at different treatment times were evaluated by real-time PCR. As observed, the degradation of the β-catenin transcript was slower in the SNHG3-OE group, while in the SNHG3-KD group it became faster (Fig. 6D). Considering that the effect of hnRNPC on RNA stability has been proved, we supposed hnRNPC to be upstream of β-catenin. The result of RIP revealed that β-catenin directly bound with hnRNPC and could be precipitated by anti-hnRNPC antibody (Fig. 6E). Taken all together, these results implicated that the overexpression of SNHG3 may enable more hnRNPC to enter the nucleus and enhance the RNA stability of β-catenin which leads to the relatively high expression of β-catenin.

### SNHG3 facilitated the metastatic ability of CRC cells by activating the Wnt/β-catenin signaling pathway

To verify the role of the Wnt/β-catenin signaling pathway in SNHG3-induced metastasis, we applied a pathway inhibitor, XAV-939, to reverse the effect of SNHG3. As shown by the transwell assay, the treatment of XAV-939 significantly reduced the number of cells that migrated through the chambers in SNHG3-OE groups including RKO and HCT 116 cell lines (Fig. 6F). In addition, the expression of EMT markers in SNHG3-OE groups, including E-cadherin, N-cadherin, Vimentin, Snail and Slug, could also be reversed after the treatment with XAV-939 (Fig. 6G). In summary, we confirmed that SNHG3 facilitates the metastasis of CRC cells through the Wnt/β-catenin signaling pathway.

### SNHG3 promoted tumor growth and peritoneal metastasis in mice and had a positive correlation with β-catenin in CRC

**Figure 7:**
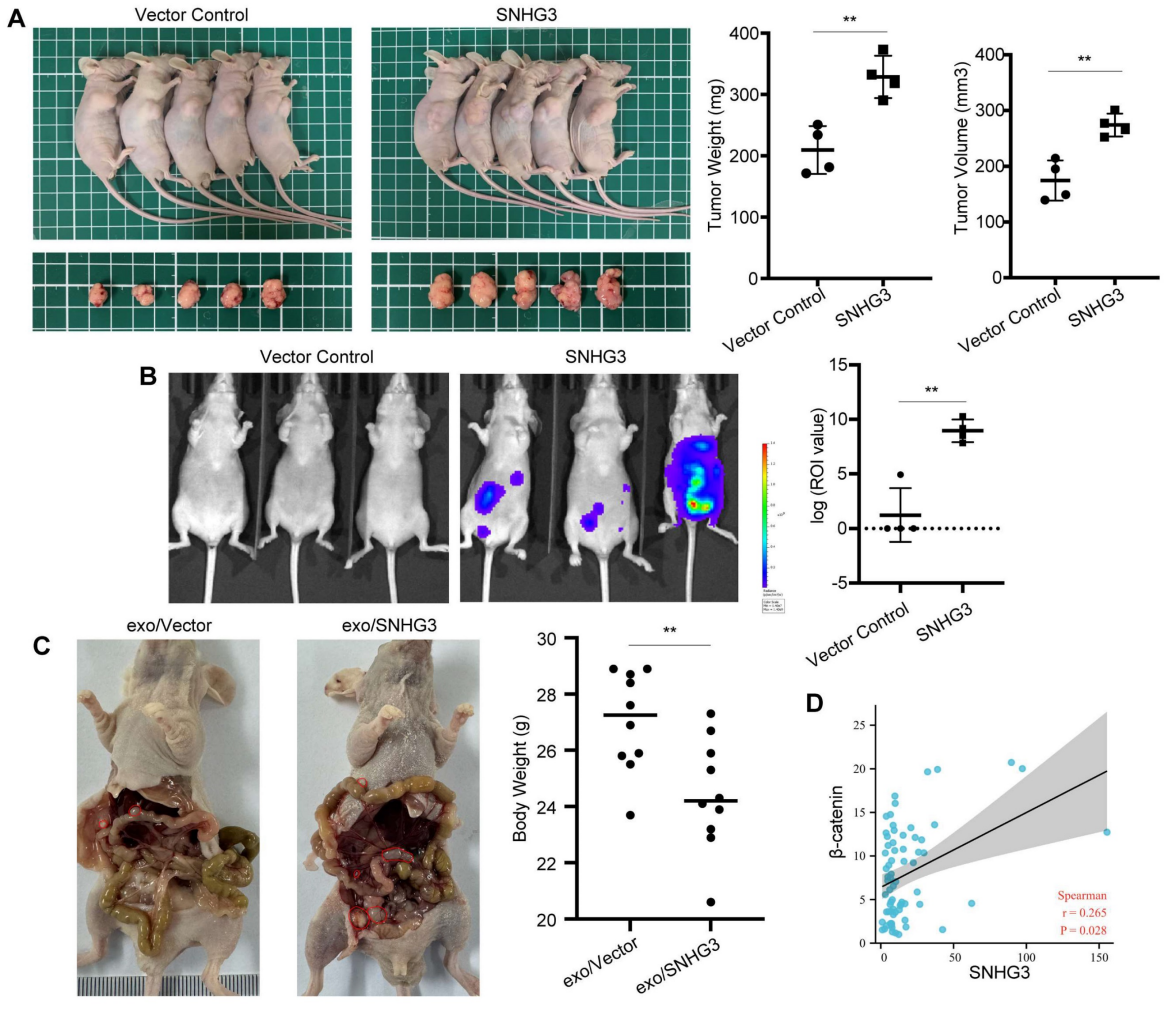
**SNHG3 promotes the proliferation and metastasis of CRC cells in vivo. A** Morphological observation of the tumor-burden nude mice at 4 weeks after the sub-axillary injection with vector or SNHG3-OE, the average volume and weight of tumors were analyzed. **B** Peritoneal metastatic model was assessed by bioluminescence imaging 4 weeks after injection, and the intensity of fluorescence was analyzed. **C** HCT 116 cells were cocultured with normal-SNHG3 or high-SNHG3 exosomes for 48h before peritoneal injection, and the body weights of nude mice were analyzed after 4 weeks. **D** Correlation analysis of SNHG3 and β-catenin in 69 collected specimens. The error bar represents the mean ± SD of three independent experiments. *p < 0.05, **p < 0.01.

We further analyzed the effect of SNHG3 on tumor growth and metastasis of CRC cells in vivo. The result of subcutaneously transplanted tumor models exhibiting more massive tumors in the SNHG3-OE group than controls confirmed the in vitro findings of SNHG3 (Fig. 7A). Moreover, as shown by the bioluminescent imaging, nude mice intraperitoneally injected with SNHG3-OE CRC cells would grow with higher tumor burdens than the control group, indicating a stronger ability of metastasis (Fig. 7B). In addition to that, the treatment of high-SNHG3 exosomes before injection facilitated the peritoneal metastasis of CRC cells, leading to lower body weights in nude mice (Fig. 7C and S5). To further examine the expression pattern of SNHG3 and β-catenin in human CRC tissues, we collected 69 specimens of frozen tissues including tumor and normal for RNA detection. As shown by the scatter plot, not surprisingly, the expression of SNHG3 exhibited a positive correlation with β-catenin (Fig. 7D). Together, these data suggested that SNHG3 can promote the tumor growth and peritoneal dissemination of CRC, and is positively correlated with the expression of β-catenin.

## Discussion

Tumor progression can be partly attributed to the interaction between malignant cells and other types of cells 27, 28. To manipulate the biological properties of recipient cells, parental cells may shed exosomes, which is a well-known mediator in intercellular communication. The widespread and steady existence of exosome in a variety of body fluids, make it an outstanding detection method for cancers 29. Exosomal lncRNAs have been reported to play an important role during the progression of CRC, some of which were identified to display great value for CRC diagnosis 30, 31. In the present study, we firstly screened the public databases and identified exosomal SNHG3 as a potential biomarker for CRC diagnosis. Our results showed that SNHG3 was overexpressed in tumor tissues of CRC patients which was consistent with previous findings 32, 33, and we also found a higher expression level of SNHG3 in serum-derived exosomes from CRC patients. These results suggested that SNHG3 may play a part in CRC progression and has the potential to be a diagnostic marker for CRC.

SNHG3 is a member of the small nucleolar RNA host genes (SNHGs) family which are the full-length transcripts from some small nucleolar RNA (snoRNA) genes containing exons and introns 34. Increasing evidence suggests that SNHG3 plays a carcinogenic role in multiple cancers, including breast cancer, osteosarcoma, hepatocellular carcinoma, gastric cancer, colorectal cancer, and so forth 33, 35-38. However, the connection between SNHG3 and exosomes is still unknown. Here, we proved the oncogenic role of SNHG3 in promoting the proliferation and migration of CRC cells, which was consistent with the previous descriptions 33, 39. Through the analysis of exosomal RNA, we confirmed that SNHG3 could be packed into exosomes by CRC cells. And the exosomes containing high levels of SNHG3 significantly induced EMT and accelerated the progression of CRC.

One of the upstream pathways of EMT is the Wnt/β-catenin pathway. It is also known as the canonical Wnt pathway and is involved in a variety of cellular processes, including proliferation, survival, apoptosis, and differentiation 40. The last stop of the canonical Wnt pathway is the accumulation of cytoplasmic β-catenin, leading to its elevated level in the nucleus which consequently initiates the expression of Wnt target genes. In the present study, we found that overexpressing SNHG3 not only activated the EMT pathway but also promoted the expression of β-catenin. Furthermore, downregulating β-catenin in SNHG3-OE CRC cells with XAV-939 rescued the promotional effect of SNHG3 and inhibited the activation of the EMT pathway. Hence, these results indicated that SNHG3 activated the Wnt/β-catenin pathway to induce EMT in CRC cells.

SNHG3 is found to exist in the nucleus as well as cytoplasm, in accordance with that, its functions are diverse 41. Most of the studies revealed that SNHG3 could work as a miRNA sponge to facilitate tumor progression. Through sponging miRNAs, such as miR-186-5p, miR-151a-3p, and miR-326, SNHG3 could release and upregulate the miRNA targets, resulting in the enhancement of proliferative, migratory, and invasive potentials of tumor cells 39, 42, 43. Further to this, Xuan et al. clarified the intranuclear role of SNHG3 in epigenetics which affected DNA methylation via binding to its corresponding methylase 37. In our study, we found that SNHG3 is mainly located in the nucleus of CRC cells suggesting the potential interaction between SNHG3 and proteins. Heterogeneous nuclear ribonucleoprotein C1/C2 (hnRNPC) belongs to a large family of RNA-binding proteins known as heterogeneous nuclear ribonucleoproteins (hnRNPs). Through recognition of single-stranded U-tracts, hnRNPC binds to a series of pre-mRNAs and modulates RNA stability, alternative splicing, and translation 44-47. By exhibiting RNA pulldown and RIP assays, we found that SNHG3 may function through binding with hnRNPC. Besides, the overexpression of SNHG3 apparently increased the intranuclear localization of hnRNPC. Based on these results, we hypothesized that SNHG3 may enhance the translocation of hnRNPC into the nucleus to promote CRC progression.

Here, our study showed that SNHG3 affected the intracellular localization of hnRNPC as well as increased the protein level of β-catenin. Therefore, we assumed that hnRNPC could be important to the upregulation of β-catenin. As expected, the result of RIP implied interaction between hnRNPC and β-catenin transcript. Considering the fact that hnRNPC has been reported to affect RNA stability, we applied ActD to examine the degradation rate of β-catenin. The result of the RNA stability assay suggested that the β-catenin transcript was stabilized when SNHG3 was overexpressed. Without a doubt, we were able to draw the conclusion that it was hnRNPC mediating the SNHG3-induced stabilization of β-catenin and enhancing its translation which consequently led to the activation of the Wnt/β-catenin signaling pathway.

In summary, our results provided evidence in support of the oncogenic role of SNHG3 in which CRC cell-secreted exosomal SNHG3 promoted metastasis of the recipient CRC cells through increasing the expression of β-catenin by enhancing its RNA stability (Fig.7). We also noticed that SNHG3 was overexpressed in serum-derived exosomes from CRC patients supporting SNHG3 as a potential marker for CRC diagnosis. However, the correlation of SNHG3 with the pathogenic characteristics and prognosis of CRC needs further investigation with more samples. This study not only expands our understanding of the oncogenic mechanisms of exosomal SNHG3 in CRC but also offers a potential marker for the noninvasive diagnostic method in CRC.

## Supplementary Material

Supplementary figures and table.

## Figures and Tables

**Figure 8:**
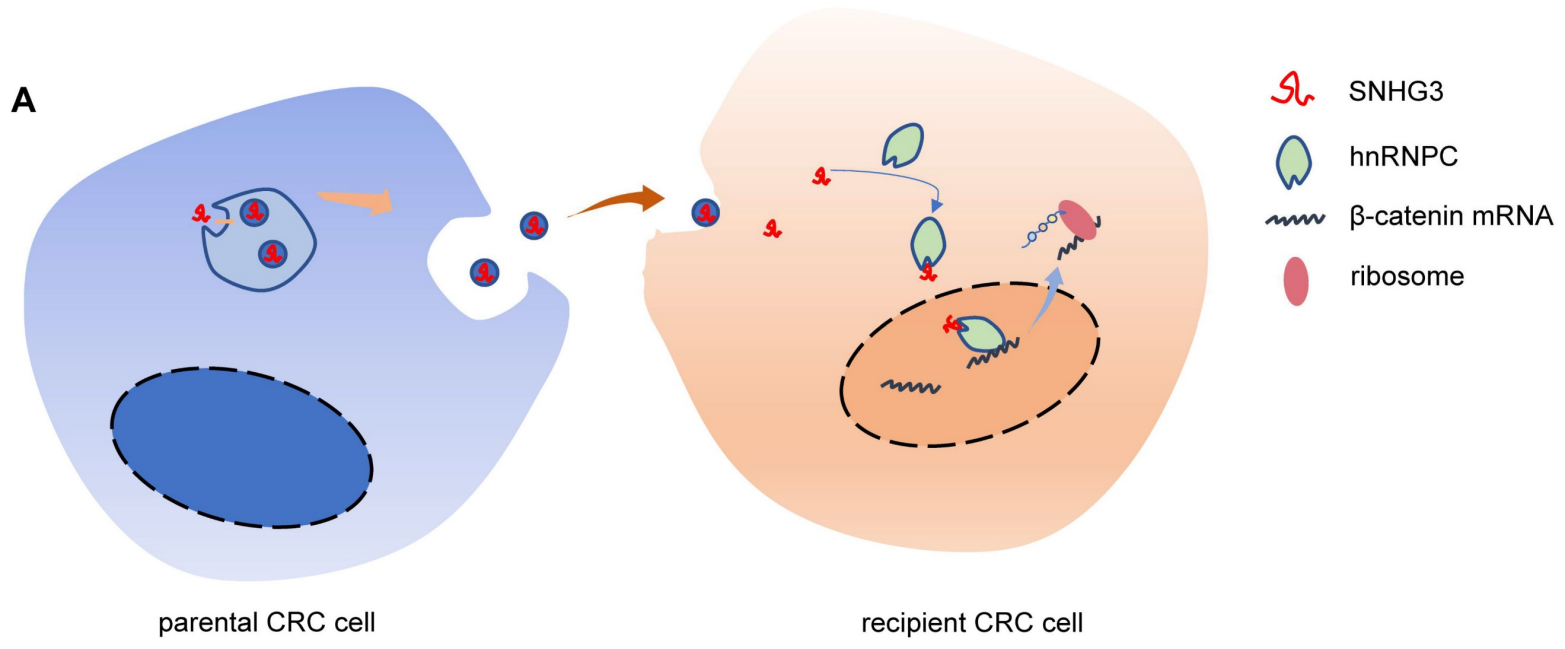
**Schematic of the mechanism by which exosomal SNHG3 was secreted to promote the metastasis of CRC.** And we revealed that SNHG3 could increase the intranuclear level of hnRNPC to enhance the stability of β-catenin and activate the EMT pathway.

## Abbreviations

CRC: colorectal cancer
lncRNAs: Long non-coding RNAs
SNHG: Small nuclear RNA host gene
EMT: Epithelial-mesenchymal transition
RIP: RNA immunoprecipitation
ActD: Actinomycin D
