# Supplementary material for: Cancer-derived exosomal lncRNA SNHG3 promotes the metastasis of colorectal cancer through hnRNPC-mediating RNA stability of β-catenin
Source: Int J Biol Sci. 2024 Apr 8;20(7):2388–402. doi: 10.7150/ijbs.88313 (PMC11077369; doi:10.7150/ijbs.88313)
Supplement: Supplementary file 1 — Supplementary figures and table. [file ijbsv20p2388s1.pdf]

Fig. S1

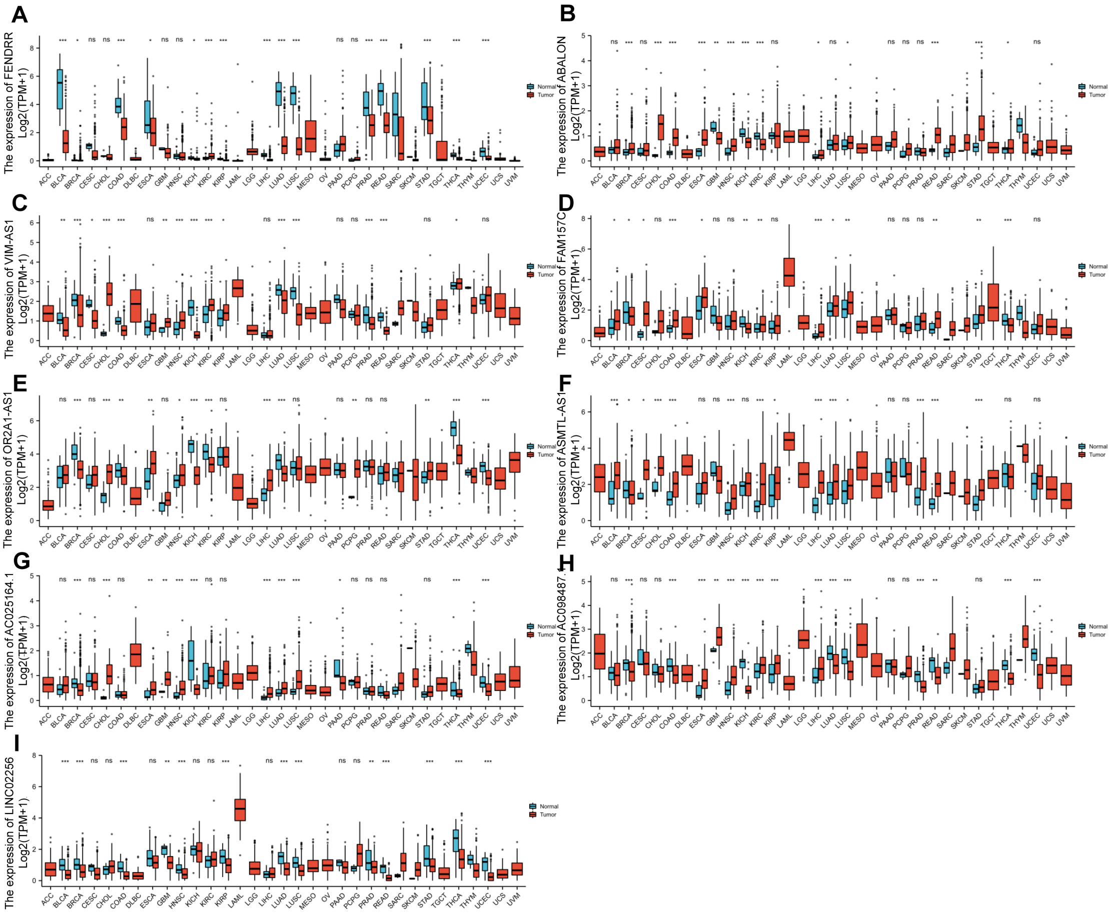
A
The expression of FENDRR
Log2(TPM+1)
B
The expression of ABALON
Log2(TPM+1)
C
The expression of VIM-AS1
Log2(TPM+1)
D
The expression of FAM157C
Log2(TPM+1)
E
The expression of OR2A1-AS1
Log2(TPM+1)
F
The expression of ASMTL-AS1
Log2(TPM+1)
G
The expression of AC025164.1
Log2(TPM+1)
H
The expression of AC098487.1
Log2(TPM+1)
I
The expression of LINC02256
Log2(TPM+1)
Normal
Tumor
ACC
BLCA
BRCA
CESC
CHOL
COAD
DLBC
ESCA
GBM
HNSC
KICH
KIRC
KIRP
LAML
LGG
LIHC
LUAD
LUSC
MESO
OV
PAAD
PCPG
PRAD
READ
SARC
SKCM
STAD
TGCT
THCA
THYM
UCEC
UCS
UVM

Fig. S2

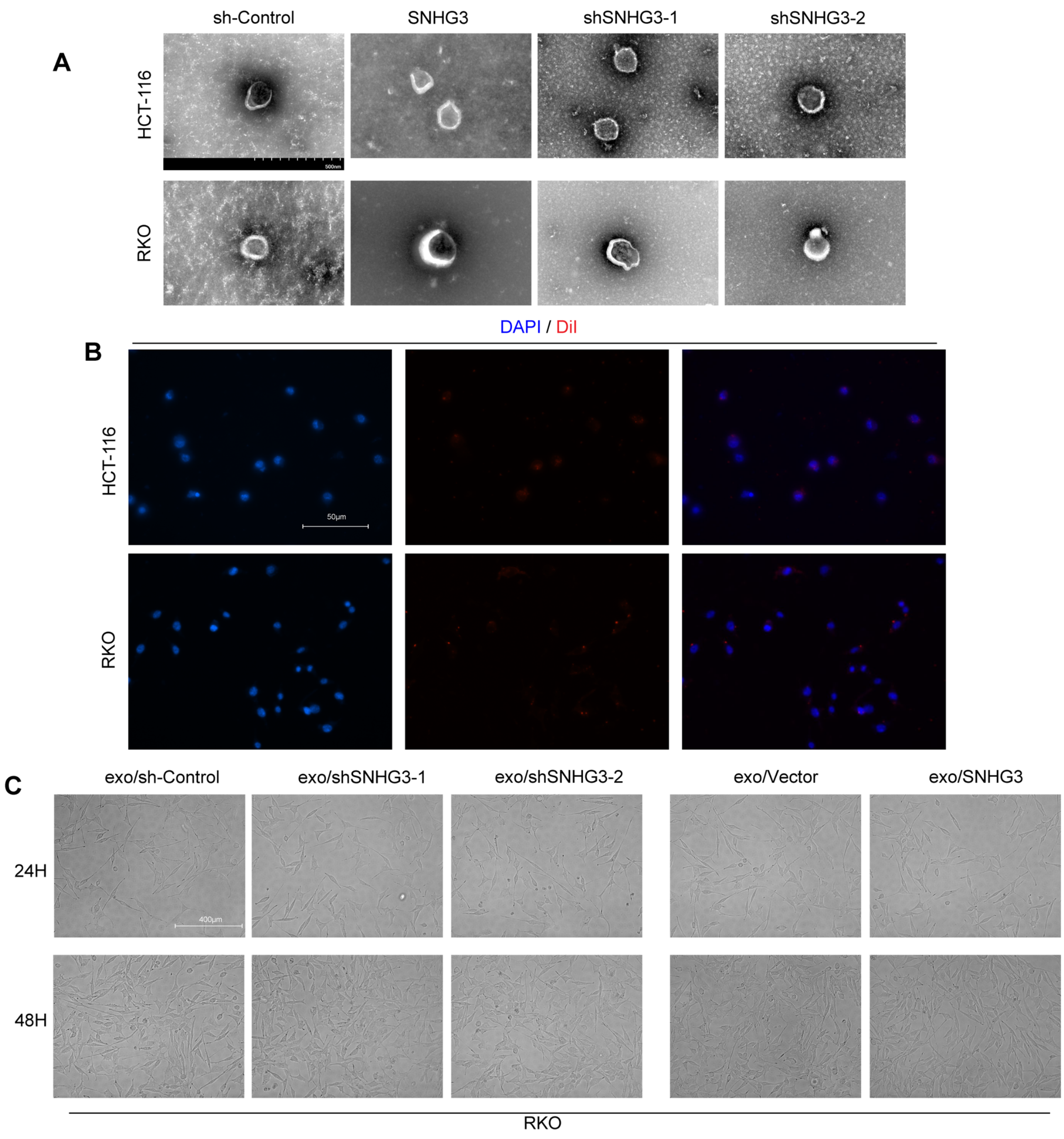
sh-Control
SNHG3
shSNHG3-1
shSNHG3-2
A
HCT-116
RKO
DAPI / Dil
B
HCT-116
50μm
RKO
C
exo/sh-Control
exo/shSNHG3-1
exo/shSNHG3-2
exo/Vector
exo/SNHG3
24H
400μm
48H
RKO

Fig. S3

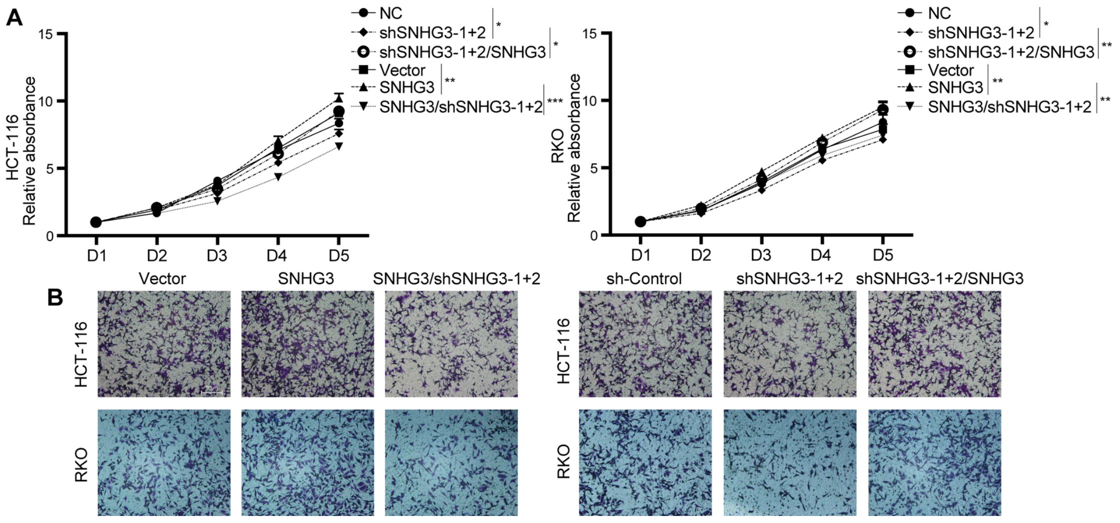
A
HCT-116
Relative absorbance
0
5
10
15
D1
D2
D3
D4
D5
NC
shSNHG3-1+2
shSNHG3-1+2/SNHG3
Vector
SNHG3
SNHG3/shSNHG3-1+2
*
*
**
***
RKO
Relative absorbance
*
**
**
**
B
Vector
SNHG3
SNHG3/shSNHG3-1+2
sh-Control
shSNHG3-1+2
shSNHG3-1+2/SNHG3
HCT-116
RKO
HCT-116
RKO

Fig. S4

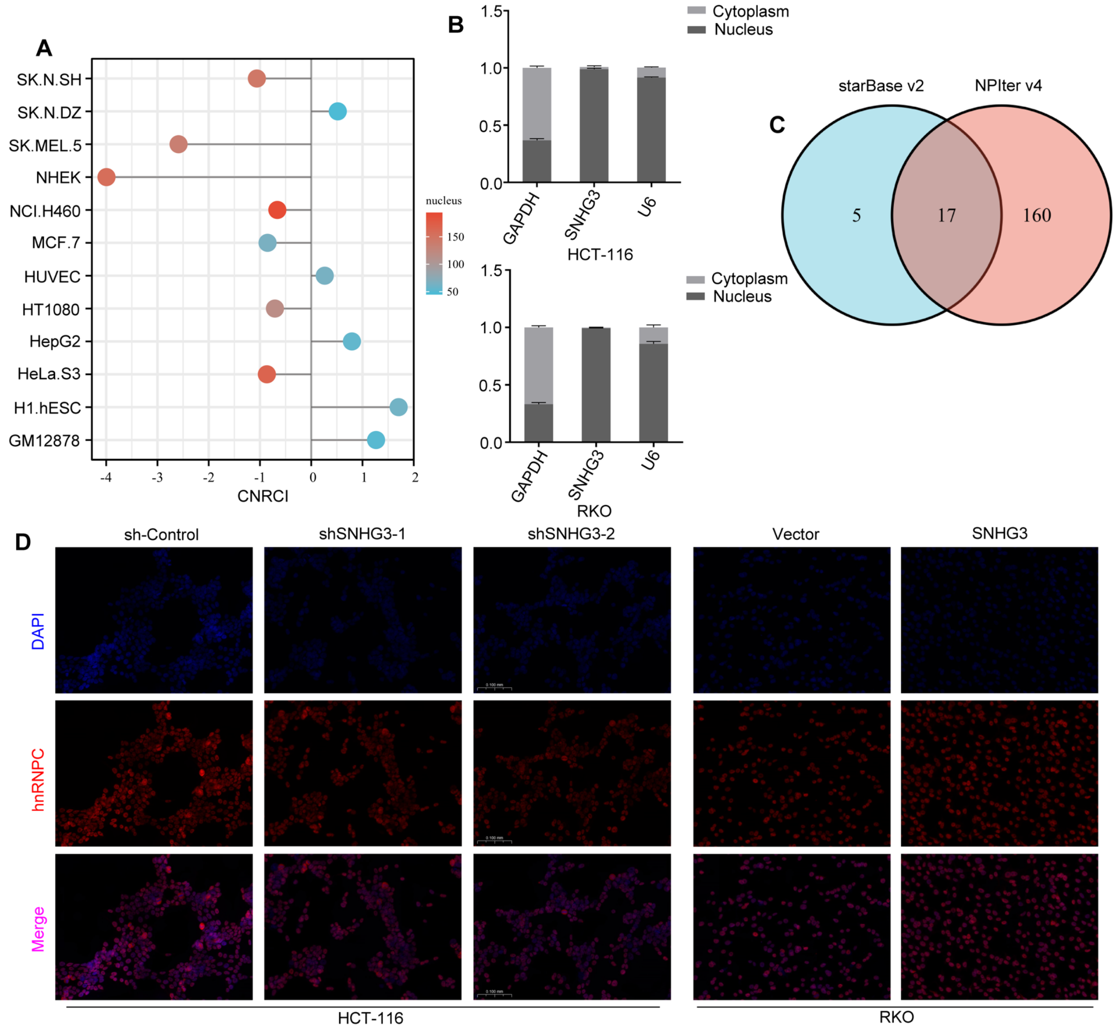

A
SK.N.SH
SK.N.DZ
SK.MEL.5
NHEK
NCI.H460
MCF.7
HUVEC
HT1080
HepG2
HeLa.S3
H1.hESC
GM12878
-4 -3 -2 -1 0 1 2
CNRCI
nucleus
150
100
50
B
Cytoplasm
Nucleus
1.5
1.0
0.5
0.0
GAPDH
SNHG3
U6
HCT-116
RKO
C
starBase v2
NPIter v4
5
17
160
D
sh-Control
shSNHG3-1
shSNHG3-2
Vector
SNHG3
DAPI
hnRNPC
Merge
HCT-116
RKO

Fig. S5

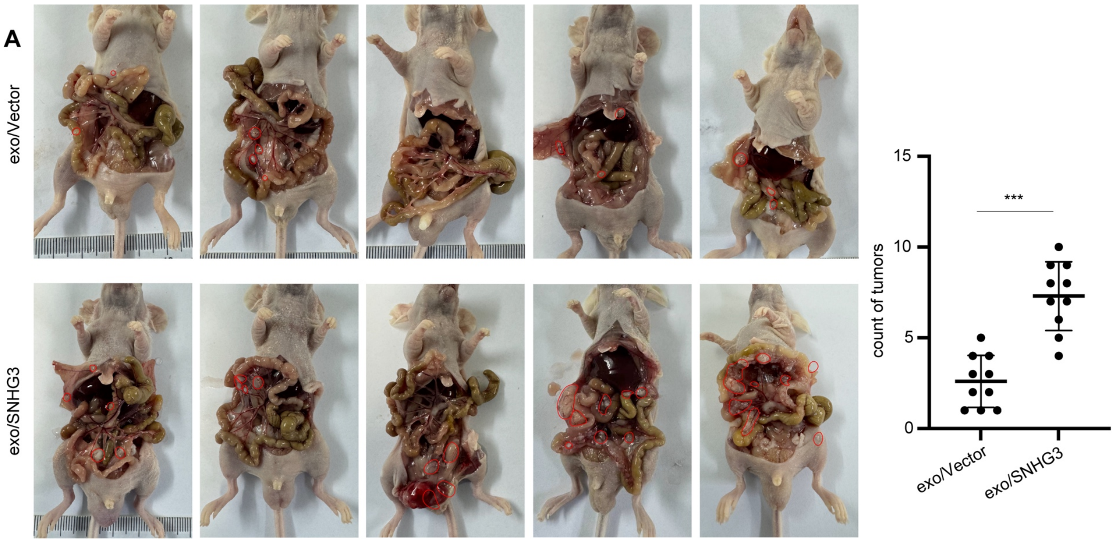
A
exo/Vector
exo/SNHG3
count of tumors
15
10
5
0
***
exo/Vector
exo/SNHG3

Table S1 Proteins predicted by starBase v2 and NPIter v4

Gene Symbol	MW (kDa)
CSTF2T	64
DDX54	99
DGCR8	100
ELAVL1	36
FMR1	71
hnRNPA1	38
hnRNPA2B1	37
hnRNPC	42
hnRNPM	77
IGF2BP1	65
IGF2BP2	66
IGF2BP3	63
LIN28B	30
RBFOX2	41
TAF15	62
TARDBP	44
U2AF2	53

Legends of supplementary figures and table

Fig. S1 The differential expression of other top 10 lncRNAs in pan-cancer. A - I TCGA pan-cancer database was applied to analyze the differential expression of FENDRR, ABALON, VIMAS1, FAM157C, OR2A1AS1, ASMTLAS1, AC025164.1, AC098487.1 and LINC02256 in multiple cancers. *$p < 0.05$, **$p < 0.01$, ***$p < 0.001$.

Fig. S2 Exosomal SNHG3 affects the morphology of recipient CRC cells. A TEM image of purified exosomes from different groups. Scale bar=500 nm. **B** The internalization of DiI-labeled exosomes secreting by RKO cells was observed using an immunofluorescent microscope. Scale bar=200 μm. C wt RKO cells were cocultured with exosomes for 24h and 48h respectively before observation.

Fig.S3 SNHG3 acts as a promoter during the progression of CRC. A and **B** CCK-8 assay and Transwell assay were applied to determine the oncogenic function of SNHG3. The error bar represents the mean ± SD of three independent experiments. *$p < 0.05$, **$p < 0.01$, ***$p < 0.001$.

Fig. S4 SNHG3 is mainly located in the nucleus of CRC cells. A lncATLAS database indicated that SNHG3 is distributed in both the nucleus and cytoplasm. **B** Real-time PCR analyzed the level of SNHG3 in different cellular fractions of CRC cells which showed a high abundance in the cytoplasm. **C** StarBase v2 and NPIter v4 were used to predict the potential proteins interacted with SNHG3, the intersection was shown by

the venn plot. **D** Immunofluorescence staining exhibited the intracellular localization of hnRNPC in CRC cells.

Fig.S5 SNHG3 promotes the peritoneal metastasis of CRC cells through exosomes. A High-SNHG3 exosomes-treated HCT 116 cells were injected to establish peritoneal metastasis models, and counts of metastatic lesions were analyzed. $*p < 0.05$, $**p < 0.01$.

Table S1 Summary of the proteins predicted to bind with SNHG3 using starBase v2 and NPIter v4. The molecular weight of each protein was listed.
